# First come, first served: neuronal processing of multi-echo streams in the auditory cortex of echolocating bats

**DOI:** 10.1242/jeb.252069

**Published:** 2026-05-28

**Authors:** M. Jerome Beetz, Manfred Kössl, Julio C. Hechavarría

**Affiliations:** ^1^ Institute for Cell Biology and Neuroscience, Goethe University, 60438 Frankfurt, Germany; ^2^ Biocenter, Department of Zoology II, Emmy-Noether Group Spatial Memory in Insects, University of Würzburg, 97074 Würzburg, Germany; ^3^ AG Brain & Behavior, Institute of Biology, Freie Universität, 14195 Berlin, Germany; ^4^ Ernst-Strüngmann-Institute for Neuroscience Frankfurt, 60528 Frankfurt am Main, Germany

**Keywords:** Echolocation, Echo delay, Biosonar, Electrophysiology, Distance coding

## Abstract

Echolocating bats emit acoustic pulses that get reflected off objects. The spatial information carried by the echoes enables bats to avoid obstacles in darkness. Usually, every pulse is followed by a cascade of echoes arising from multiple objects. By using echolocation sequences where single pulses are followed by echo cascades, we recently demonstrated that cortical neurons predominantly responded to the leading echo. Responses to lagging echoes from a cascade were suppressed, suggesting that spatial information from the most immediate object is processed at the cortex level. In that study, the leading echo was typically the most intense, leaving it unclear whether the echo selectivity was due to echo order or echo level. Here, we recorded from the auditory cortex of anaesthetized *Carollia perspicillata*, while stimulating the bats with echolocation sequences that contained echo cascades either with echo levels that were equally intense or where the leading echo was less intense than the lagging ones. Our results demonstrate that the echo level has only minor effects on neural processing and that the echo selectivity is mostly caused by the echo order. These results go in line with the neural time window of sensation hypothesis, proposed by Roverud and Grinnell. Whenever the bat hears a pulse, a neural time window opens, and any subsequent high-frequency signal within the spectral range of that pulse is by default classified as an echo, thereby closing the sensation window. This mechanism renders large parts of the cortex less responsive to distant objects, regardless of the echo intensity they produced.

## Teaser

**Summary:** Cortical neurons tuned to object distance predominantly respond to the leading echo of a multi-echo cascade in the echolocating bat *Carollia perspicillata*.

## INTRODUCTION

Echolocation allows bats to navigate in darkness. To this end, they broadcast high-frequency pulses and extract spatial information from returning echoes (Beetz and Hechavarría, 2022; Moss and Surlykke, 2010; Neuweiler, 1990). To rapidly negotiate obstacles during flight, bats must continuously monitor their distances to objects. Because sound waves travel at constant speed in a given medium, the time between pulse emission and echo arrival, i.e. the echo delay, is proportional to the target distance. For example, an echo delay of 10 ms is equivalent to an object's distance of 1.7 m. In the bat nervous system, neurons tuned to echo delays have been extensively studied by presenting a pulse that is followed by a single echo (Feng et al., 1978; Grinnell, 1963; Hagemann et al., 2011; Suga and O'Neill, 1979; Sullivan, 1982). This stimulus design represents a strongly simplified version of the acoustic world that a bat may encounter. For example, in cluttered environments, each pulse is reflected off multiple objects, which results in a cascade of successive echoes representing objects located at different depths in space relative to the calling bat (Moss and Surlykke, 2010). To keep track of every object, bats must process multiple object-related auditory streams in parallel, a computationally demanding task. Although motor adjustments such as pinpointing the sonar beam or adjusting the highly mobile outer ears to objects of interest may reduce the number of received echoes (Fujioka et al., 2014; Gao et al., 2011; Seibert et al., 2013; Surlykke et al., 2009a,b; Wohlgemuth et al., 2016), these motor adaptations are insufficient to avoid echo cascades (Linnenschmidt and Wiegrebe, 2016). Behavioural experiments in which *Noctilio albiventris* was trained in a distance discrimination task revealed that extracting object distances is severely degraded when high-frequency signals were presented between pulse and echo (Roverud and Grinnell, 1985). Based on these behavioural results, the authors proposed a ‘neuronal time window of sensation’. According to this neural filter, a ‘time window of sensation’ opens whenever the bat broadcasts a pulse. A subsequent high-frequency signal that falls within the spectral range of the bat's echolocation signal is automatically classified as the echo and closes the time window. This neural filter may ensure correct pulse–echo assignments even when the bat flies in highly cluttered environments where echo cascades are unavoidable.

The ‘neuronal time window of sensation’ is also supported by physiological findings from the auditory cortex and auditory midbrain (Beetz et al., 2018). Delay tuning was most severely perturbed when an acoustic interferer, i.e. an echolocation pulse, was presented in the first half of a naturalistic echolocation sequence of an approach flight. This result was explained by the fact that long echo delays were exclusively presented in the first half of the sequence, which increases the likelihood of an acoustic interferer occurring between a pulse–echo pair. In another study, we presented an echolocation sequence that contained echo cascades (multi-object sequence) and showed that cortical neurons predominantly respond to echoes from the most immediate object, represented by the leading echo of a cascade (Beetz et al., 2016a). Lagging echoes from distant objects were not processed at the cortical level. These physiological findings are in line with the time window of sensation, which, as mentioned above, opens whenever the bat hears a high-frequency signal, i.e. the pulse, and closes at the arrival of the first echo or after ∼25 ms of silence (Roverud and Grinnell, 1985).

So far, studies investigating neuronal processing of echo cascades have not explicitly tested the influence of echo levels, i.e. echoes from the closest object were typically most intense whereas subsequent echoes from distant objects were fainter (Beetz et al., 2016a, 2017; Greiter and Firzlaff, 2017). With this stimulus design, it is not possible to test whether the neurons' selectivity to echoes from the most immediate object is due to the echo order or to the echo intensity. Because crashing into an object – irrespective of its size – may be of potential danger for a flying bat, we hypothesized that the neuronal time window of sensation is intensity independent and ensures that echoes from the closest object are predominantly processed, even if these echoes are fainter than lagging ones. In this article, we tested the above-mentioned hypothesis by recording the activity of cortical neurons while anaesthetized bats heard multi-echo streams in which lagging echoes were louder than leading echoes. The results show that indeed, most cortical neurons respond to leading echoes irrespective of the intensity of lagging sounds.

## MATERIALS AND METHODS

### Animals

Experiments were conducted in 9 adult female bats of the species *Carollia perspicillata* (Linnaeus 1758). The bats were bred and reared in a bat colony of the Institute for Cell Biology and Neuroscience (Frankfurt University). The animal use in the experiments complies with all current German laws on animal experimentation. All experimental protocols were approved by the Regierungspräsidium Darmstadt (experimental permit no. F104/57).

### Acoustic stimulation

**Fig. 1.**
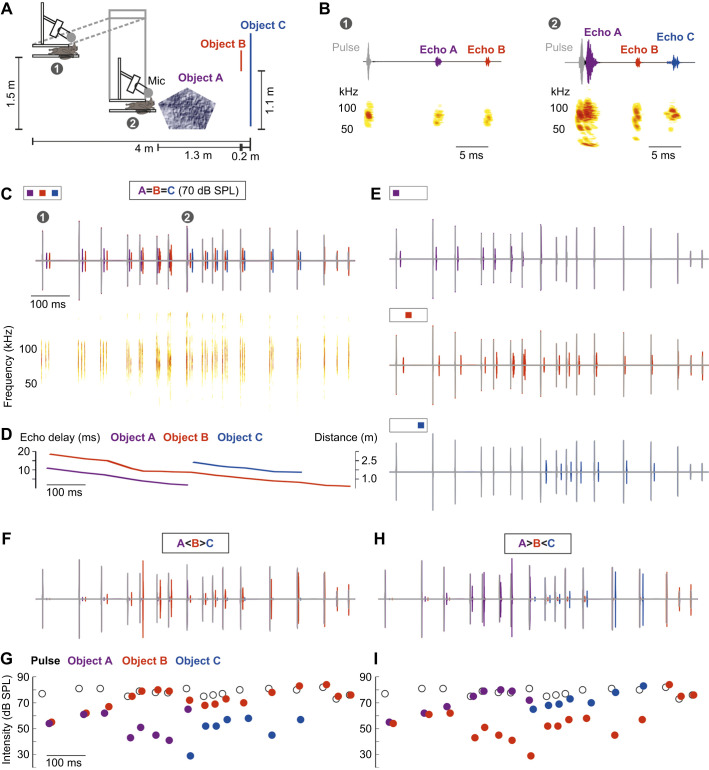
**Acoustic stimuli used for neuronal recordings.** (A) Scheme showing the pendulum used to record echolocation sequences. Distances between objects (object A, object B, object C) were set so that returning echoes did not overlap in time. (B) Oscillogram and spectrogram of two exemplary pulse–echo cascades. Numbers refer to the approximate pendulum position when these acoustic signals were recorded (see A). Every pulse was followed by two to three echoes. (C) Oscillogram and spectrogram of the multi-object sequence (icon in the upper left corner signals the objects represented in the sequence). Echo levels were set to 70 dB SPL. (D) Echo delay/object distance as a function of time. (E) The multi-object sequence was modified to single-object sequences by deleting object-specific echoes. (F) Oscillogram of the multi-object sequence whose object B echoes were most intense within the echo cascades. (G) Scatterplot of pulse and echo levels for the sequence shown in F. (H) Oscillogram of the multi-object sequence whose object B echoes were least intense within the echo cascades. (I) Scatterplot of pulse and echo levels for the sequence shown in H.

The acoustic stimuli were modified versions of the naturalistic echolocation sequence that was used in earlier studies (Beetz et al., 2016a, 2017). The echolocation sequence was recorded from a bat that was placed in the mass of a pendulum (Fig. 1A). Along the pendulum's swing trajectory, three objects were placed at different distances. Object A was a dummy rock (depth: 65 cm; width: 95 cm; height: 35 cm) made out of papier-mâché and it was passed in the middle of the swing before the pendulum stopped in front of object B, a wooden plate (depth: 0.8 cm; width: 21 cm; height: 21 cm) positioned 130 cm behind object A and 20 cm in front of object C. Object C, a transparent acrylic wall (depth: 0.3 cm; width: 50 cm; height: 150 cm). During the swing, the bat broadcasted echolocation pulses that were, together with echoes, recorded with an ultrasound sensitive microphone (Avisoft Bioacoustics, Glienicke/Nordbahn, Germany). The microphone was attached to the pendulum so that it was medially positioned above the bat's head and as close as possible to the bat's ears (∼4 cm). It had a sensitivity of 50 mV Pa^−1^ and an input-referred self-noise level of 18 dB SPL. A sound acquisition system (UltraSoundGate 116Hm mobile recording interface, +Recorder Software, Avisoft Bioacoustics) recorded the echolocation signals at a sampling rate of 375 kHz (16-bit precision) and later resampled to 384 kHz. The distance between objects was large enough that the echoes from each object were non-overlapping in the echo stream (Fig. 1B). Each pulse was followed by at least two echoes, each coming from a different object.

Before using the echolocation sequence as an acoustic stimulus for neural recordings, we filtered background noise of the audio recording (FFT length 256; precision 16) in the software Avisoft SAS Lab Pro (Avisoft Bioacoustics) as described in Beetz et al. (2016b). To control for the echo level, we adjusted the intensity so that each echo was equally intense (70 dB SPL). As echoes were non-overlapping along the time axis, we could simply delete object-specific echoes in the software BatSound (PetterssonElektronik AB, Uppsala, Sweden). This allowed us to transform the ‘multi-object sequence’ (Fig. 1C,D) into three ‘single-object sequences’, each containing echoes from one of the three objects (Fig. 1E). From the original echolocation sequence, we swapped the intensity of object-specific echoes so that echoes from object B were always the most intense (Fig. 1F,G) or the least intense (Fig. 1H,I).

During the neural recordings from anaesthetized bats, acoustic stimuli were played at a sampling rate of 384 kHz (32-bit precision) with an Exasound E18 sound card (ExaSound Audio Design, Toronto, ON, Canada) connected to an audio amplifier (Rotel power amplifier, RB-850). The speaker (ScanSpeakRevelator R2904/7000, Avisoft Bioacoustics) was placed 15 cm from the bat's contralateral ear (in relation to the electrode position). A speaker-specific calibration curve was calculated with a ¼-inch Microphone (Brüel&Kjaer, model 4135, Nærum, Denmark) and a custom-made microphone amplifier. Each acoustic stimulus, i.e. echolocation sequence, was played 15 times in a pseudo-randomized order. An inter-sequence time interval of 400 ms was used to allow neurons to recover from neuronal suppression that is typically induced by the acoustic rate of the echolocation sequence (Beetz et al., 2016b; Martin et al., 2017).

### Neuronal recordings

For anaesthesia, bats were subcutaneously injected with a mixture of ketamine (10 mg kg^−1^ Ketavet, Pharmacia GmbH, Berlin, Germany) and xylazine (38 mg kg^−1^ Rompun, Bayer Vital GmbH, Leverkusen, Germany). Surgery and chronical recordings were conducted from both brain hemispheres as described in Beetz et al. (2016b).

Recordings were performed in a sound-proofed Faraday cage with either commercial electrode arrays (Tucker Davis Technologies, Omnetics Based Microwire Arrays) consisting of two rows, each row with eight electrodes and 250 μm distance between each electrode, and an inter-row space set to 500 μm (for three recordings; two times from f22, once from each brain hemisphere; and once from f26), or custom-built glass electrode arrays consisting of six (for five recordings; f19, f20, f21, f22, f25) or eight (for three recordings; f28, f29, f30) electrodes. Glass electrodes (resistance 1–10 MΩ when filled with 3 mol l^−1^ KCl) were drawn from borosilicate glass capillaries (GB120F-10, Science Products, Hofheim, Germany) with a Flaming/Brown horizontal puller (P97, Sutter Instrument, Novato, CA, USA), and were glued together in a fan-shaped pattern. The space between the electrode tips was set to 250 μm under the microscope. Electrode penetration occurred with the dura mater intact. The electrode array was positioned along the chronotopic gradient (Hagemann et al., 2010) in the high-frequency area of the auditory cortex (Esser and Eiermann, 1999). A silver wire placed between the skull and dura mater served as a reference.

For data acquisition, we used a wireless multichannel recording system (Multi Channel Systems MCS GmbH, Reutlingen, Germany) at a sampling rate of 20 kHz (per channel) and 16-bit precision. MCS files were imported into Spike2 (Version 9.0, Cambridge Electronic Design) where multi-unit specific spike thresholds were set manually. Neural signals surpassing this threshold were defined as spike events and their time point exported as a mat-file. Spike events were further analysed in custom-written scripts in Matlab (R2021a). Altogether, 76 multi-units, each sensitive to high frequencies were analysed for this study. To measure frequency tuning, we presented 2 ms pure tones that varied between 5 and 95 kHz (5 kHz intervals) and 30 and 90 dB SPL. Sound levels were adjusted based on the speaker's calibration curve. Each frequency-level combination was randomly presented five times with a 400 ms interstimulus time interval.

### Analysis

Neural data were analysed based on post-stimulus time histograms (PSTHs; 5 ms bin size) computed for each multi-unit. To compute a multi-unit's best delay, i.e. the echo delay evoking the strongest response, we identified the highest bin of the PSTH measured in response to the single-object sequence containing echo information of object B and defined the echo delay preceding this bin as the multi-unit's best delay. We selected this single-object sequence because a broad range of echo delays (2–20 ms) was presented. The initial 75 ms of the PSTH were not considered for analysis because many multi-units show a strong onset response to our acoustic stimuli. These onset responses are common and do not represent delay tuning because they also occur in the absence of echoes, i.e. only when pulses were presented (Beetz et al., 2016b).

To compare the neural responses elicited by the multi-object sequence and the single-object sequences, we correlated the PSTHs with each other for every multi-unit. As echoes from object A were only present at the first 450 ms of the multi-object sequence, we computed correlation values for the first and second half of the PSTHs separately. With the correlation values, we quantified which object was preferentially represented in the neural response to the multi-object sequence. For comprehensibility, we labelled the correlation values as object preference indices.

To analyse the effect of the leading echo on the neural response to the lagging echo, we grouped the bins of the PSTHs to the corresponding echo cascade and referred the plot to as ‘activity curves’. This allowed us to compute correlation values cascade-wise with the corresponding echo of each single-object sequence. Additionally, we predicted the neural response to the leading echo of a cascade by considering the neural response to the single-object sequence representing the leading echo. For example, we calculated the number of spikes evoked by the single-object sequence representing echoes from object A. This predicted response strength to object A was then compared with the neural suppression observed in response to the multi-object sequence. The suppression rate was calculated by counting the number of spikes in response to the multi-object sequence and dividing it by the number of spikes predicted from responses to the single-object sequences. This calculation was done for object A and object B in the first half of the sequence and for object B and object C in the second half of the sequence. This separation was necessary because object B was the source for the lagging echo in the first half of the sequence, while echoes from object B were leading the cascades in the second half of the sequence.

The predicted response strength to the leading echo was also compared with the correlation value obtained by comparing the neural response to the single-object sequence of the lagging echo and the multi-object sequence. Here again, two values were computed for each multi-unit, for the first and the second half of the sequence.

Graphs were computed either in Matlab (R2021a) or in GraphPad Prism 10 (GraphPad Software).

### Statistics

Statistics were done in GraphPad Prism 10 (GraphPad Software). First, data were tested for a normal distribution with a D'Agostino and Pearson test. Correlation values showed no normal distribution (alpha=0.05) and were statistically compared with a Friedman test (paired) followed by a Dunn's multiple comparisons *post hoc* test. A Mann–Whitney test was used to compare statistically the preference to process the leading or lagging echo and the best delay. Correlation between the normalized response to the leading echo and the suppression rate to the response to the lagging echo of a cascade was quantified with a two-tailed Spearman test. Correlations between the normalized response to the leading echo and the preference index to the lagging echo of a cascade was also quantified with a two-tailed Spearman test. A two-tailed Spearman test was also used to test for a correlation between the best delay and the preference index.

## RESULTS

**Fig. 2.**
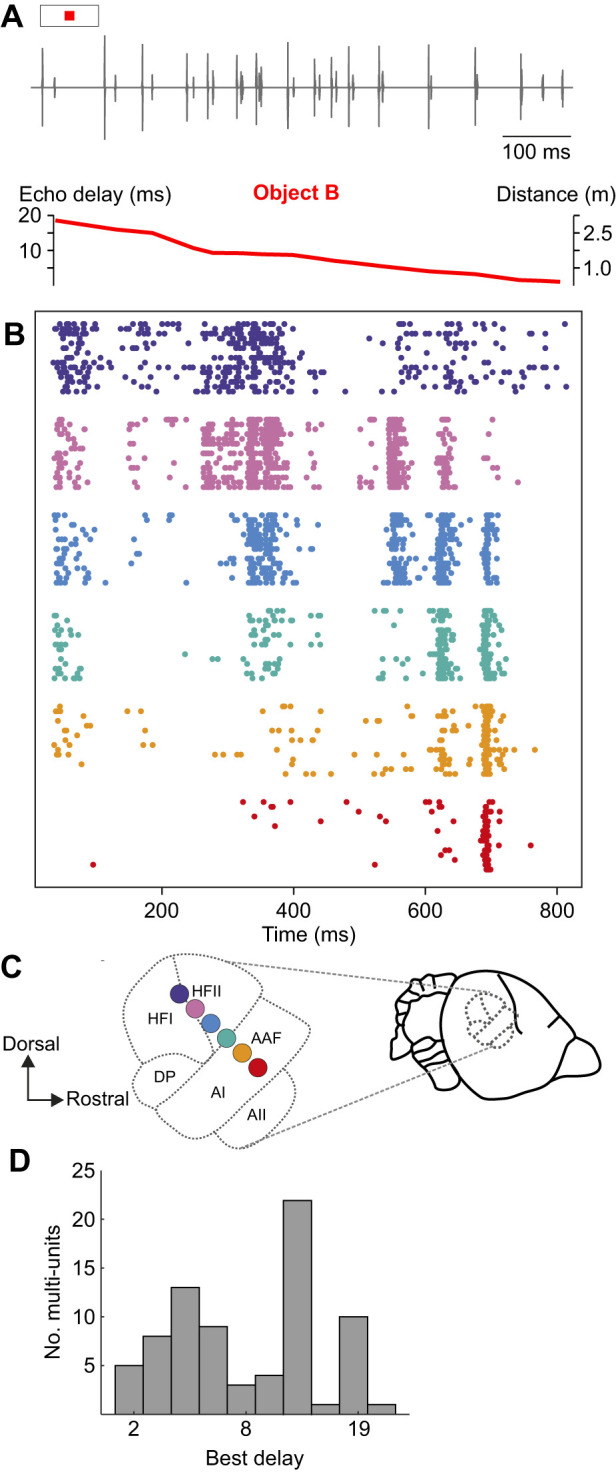
**Characterization of delay tuning in the auditory cortex of *Carollia perspicillata*.** (A) Top: oscillogram of the single-object sequence that contains echoes from object B. Bottom: echo delay/object distance over time. (B) Raster plots showing the neural response of six colour-coded multi-units to 15 presentations of the single-object sequence. Each dot represents an action potential. The multi-units were simultaneously recorded and ordered along the rostral–caudal axis of the bat's cortex. Notably, caudal multi-units (upper raster plots) tend to respond to earlier sequence portions than rostral multi-units (lower raster plots). (C) Schematic side view of the brain of *C. perspicillata*. The auditory cortex is highlighted and enlarged on the left side. An electrode array of six electrodes was placed along the rostral–caudal axis of the auditory cortex. The colours code for the multi-unit responses shown in B. AAF, anterior auditory field; AI, primary auditory cortex; AII, secondary auditory cortex; DP, dorsoposterior field; HF, high-frequency area. (D) Histogram of best delays for 76 multi-units measured in response to the single-object sequence.

We recorded from 76 multi-units of the auditory cortex of nine anaesthetized bats (6.91±1.93 multi-units per recording). Every multi-unit was sensitive to high frequencies and responded to certain portions of the echolocation sequence containing echoes from object B only (Fig. 2A). To calculate a multi-unit's best delay, we searched for the time point of the echolocation sequence that evoked the strongest response and assigned the echo delay preceding the response to the best delay (Fig. 2A). Echo delays of the echolocation sequence used for calculating the best delay ranged from 2 to 20 ms. Placing an electrode array along the rostral–caudal axis of the auditory cortex revealed a chronotopic gradient of delay tuning (Fig. 2B,C). Dorsal–caudal neurons (purple and pink units in Fig. 2B) responded most strongly to the first half of the sequence that represented mid-length to long echo delays (10–20 ms). Contrastingly, neurons of the ventral–rostral part of the cortex (orange and red units in Fig. 2B) responded most strongly to short echo delays (2–4 ms) represented at late portions of the sequence. A chronotopic organization of best delays along the rostral–caudal axis of the cortex was observed in many of the recordings (Fig. S1). However, because neurons tuned to short delays are distributed all over the rostral–caudal axis, some chronotopic maps were blurry, as reported in an earlier study (Hechavarría et al., 2013).

About 39.5% of the multi-units (*n*=30) were tuned to echo delays between 4 and 7 ms (Fig. 2D). This bias in delay tuning was independent of the echo level, as similar trends were observed when varying the pulse–echo levels (Fig. S2). An overrepresentation of mid-length echo delays has also been reported with single-unit recordings and classical stimulation protocols that used single pulse–echo pairs (Hagemann et al., 2011; Hechavarría et al., 2013).

### Neuronal responses to echo cascades at constant echo levels

In a previous study, we observed that cortical neurons respond more strongly to the leading echo of an echo cascade (Beetz et al., 2016a), when stimulating anaesthetized bats with a multi-object sequence where each pulse was followed by two to three echoes (echo cascades). However, in that study, as well as in a study from a different laboratory (Greiter and Firzlaff, 2017), the echo levels within a cascade varied so that the first echo was typically most intense. To test whether the neuronal preference of responding to the leading echo is due to its sound pressure level or to its order within a cascade, in the present study we presented a multi-object sequence to the bat whose echo levels were constant at 70 dB SPL within a cascade (Fig. 1C). To quantify which echo of a cascade evokes the strongest response, we additionally presented three single-object sequences (Fig. 1E), where each pulse was followed by one echo only. To ensure that, except for the absence of object-specific echoes, the single-object sequences were identical to the multi-object sequence, the single-object sequences were created by deleting object-specific echoes from the original multi-object sequence. This was only possible because the echoes were non-overlapping as a result of the distances between the objects. Because the bat passed object A in the middle of the swing (at ∼400 ms; Fig. 1A), the order of object B echoes changed during the swing. During the first half of the sequence, echoes from object B were in the second place within a cascade. Because of passing object A, echoes from object B became the leading echoes in the second half of the sequence. This allowed us to explicitly test whether a change in the echo order of object B affects the neural tuning. According to our previous study (Beetz et al., 2016a), we hypothesized that echoes from object B were only predominantly processed in the second half of the sequence when they were the leading echoes in the echo cascades. In contrast to this, in the first half of the sequence where echoes from object A were leading, we hypothesized that object A was predominantly processed.

**Fig. 3.**
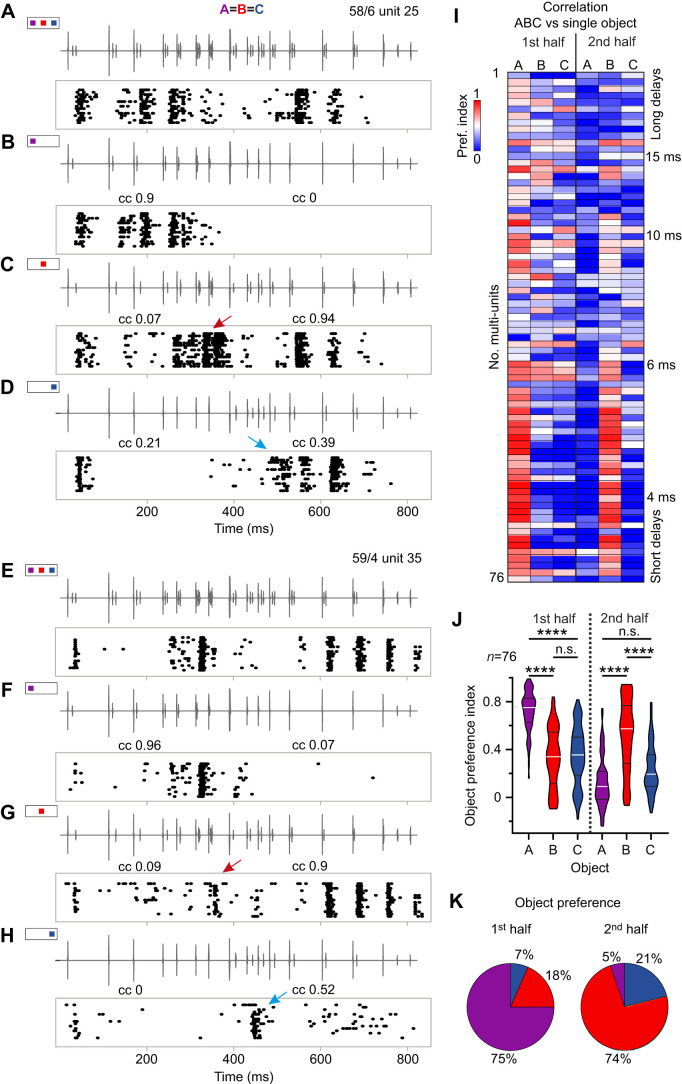
**Neural responses to echo cascades of equal intensity.** (A–H) Top: oscillograms of the acoustic stimuli. Schemes in the upper left corner indicate the objects whose echoes were present in the echolocation sequence. Bottom: raster plots visualizing the neural response of two multi-units (unit 25: A–D; unit 35: E–H; with experiment ID/electrode ID shown to the left). Correlation values (cc) obtained by comparing the neural responses with the single-object sequences and the multi-object sequence are indicated. Correlation values were separately computed for the responses to the first and second half of the sequence. Red and blue arrows indicate the neural response that was suppressed in the multi-object sequence but that was present in the response to the single-object sequence. (I) Colour-coded correlation values (preference indices) from the neural response to every single-object sequence (object A, B, C) and the multi-object sequence for the first and second half of the sequences. Multi-units are ordered according to their best delay. (J) Violin plots summarizing the object preference indices from 76 multi-units. In the first half of the sequence, the neural response to the multi-object sequence mostly resembles the response to object A. In the second half of the sequence, the neural response to the multiple-object sequence is mostly dictated by echoes from object B. Friedman test: ****P<10^−5^, Friedman statistic=162.9, *n*=76; Dunn's multiple comparisons test: object A versus B first half: ****P<10^−5^, *Z*=7.11; A versus C first half: ****P<10^−5^, *Z*=7.11; B versus C first half: *P*>0.9999, *Z*=0; A versus B second half: ****P<10^−5^, *Z*=6.42; A versus C second half: *P*>0.9999, *Z*=1.69; B versus C second half: ****P<10^−5^, *Z*=4.73. (K) Pie charts visualizing the percentage of object preferences for the first half (left) and the second half of the sequence (right) (*n*=76 multi-units).

In line with our hypotheses, the neural responses to the first half of the multi-object sequence mostly resembled the responses to the single-object sequence that contained echoes from object A (Fig. 3A–H). Correlation values (preference indices) calculated from the neural responses to the first half of the sequences were highest for object A in most multi-units (Fig. 3I). Neuronal responses to object B that were predicted based on the response to the single-object sequence were suppressed in the multi-object sequence (red arrows in Fig. 3C,G and Fig. S3).

In the second half of the multi-object sequence, correlation values were highest between the responses to the single-object sequence containing echoes from object B and the multi-object sequence (Fig. 3A–J). Notably, neuronal responses to lagging echoes could also be suppressed even when no spikes were elicited by the leading echo (Compare Fig. 3A with blue arrows in Fig. 3D,H).

**Fig. 4.**
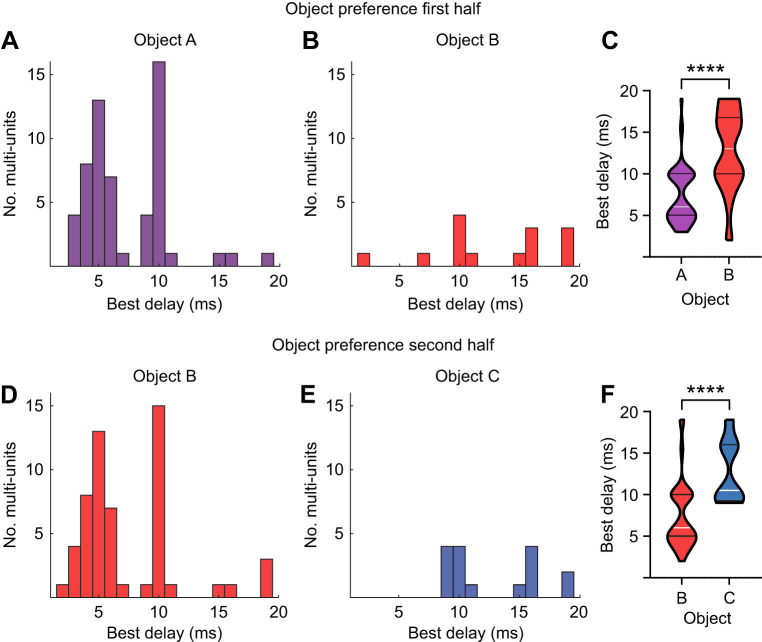
**Correlation between best delay and object preference.** (A,B) Distribution of best delays for multi-units with maximum preference indices to object A (A) and object (B) in the first half of the sequence. (C) Violin plots showing the best delays for multi-units preferentially responding to object A and object B in the first half of the sequence. Mann–Whitney test: ****P<10^−5^, Mann–Whitney *U*=141.5, *n*=76 multi-units. (D,E) Distribution of best delays for multi-units with maximum preference indices to object B (D) and object C (E) in the second half of the sequence. (F) Violin plots showing the best delays for multi-units preferentially responding to object B and object C in the second half of the sequence. Mann–Whitney test: ****P<10^−5^, Mann–Whitney *U*=167, *n*=76 multi-units.

Although most multi-units preferentially responded to the first echo of a cascade, some multi-units showed highest preference indices for the second echo (18% and 21% for the first and second half of the sequence, respectively) or even the third echo (7%; Fig. 3K). In particular, multi-units with best delays longer than 10 ms tended to respond less robustly to the first echo, represented by cool colours for object A in the first half of the sequence and object B in the second half of the sequence in Fig. 3I. Splitting the data into multi-units that preferentially responded to the first echo or the second echo confirmed the observation that multi-units preferentially responding to the second echo, i.e. object B and object C for, respectively, the first and second half of the sequence, had best delays longer than 9 ms (Fig. 4). In line with this result, the preference index to echoes of the most immediate object negatively correlated with the multi-units' best delays, i.e. the longer the best delay, the lower the preference index to encode the most immediate object (Spearman: *r*=−0.57, *P*<10^−5^, *n*=76 multi-units; Fig. S4A; Spearman: *r*=−0.56, *P*<10^−5^, *n*=76 multi-units; Fig. S4E). This correlation was reversed when plotting the preference indices of lagging echoes against the multi-units' best delays (Spearman: *r*=0.2, *P*=0.0445, *n*=76 multi-units; Fig. S4B; Spearman: *r*=0.29, *P*=0.0058, *n*=76 multi-units; Fig. S4C; Spearman: *r*=0.37, *P*=0.0004, *n*=76 multi-units; Fig. S4F).

**Fig. 5.**
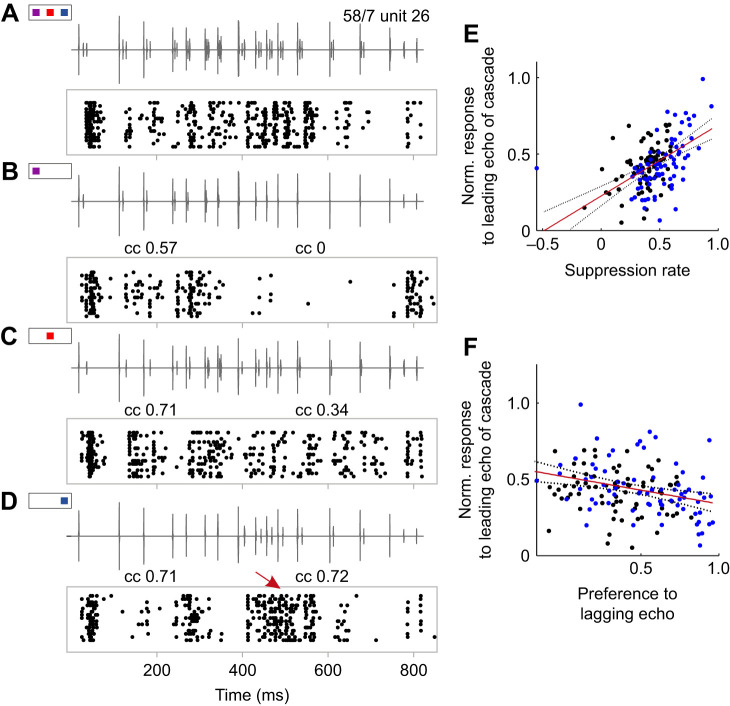
**Response strength to the first echo correlates with neural suppression to the lagging echo.** (A–D) Top: oscillograms of the acoustic stimuli. Schemes in the upper left corner indicate the objects whose echoes were represented in the echolocation sequence. Bottom: raster plots visualizing the neural response of one multi-unit. Correlation values (cc) were computed by comparing the neural response to one of the single-object sequences with the response to the multi-object sequence. Correlation values were separately computed for the responses to the first and second half of the sequence. Notably, the multi-unit responded poorly to object B in the second half of the sequence (C). Red arrow signals the neural response to object C, which was not suppressed because of the presence of echoes from object B in the response to the multi-object sequence (A). (E) Linear correlation between the normalized response to the first echo and the suppression rate to the second echo of a cascade. Spearman: *r*=0.4998, ****P<10^−5^, *n*=152 (76 data points from the first half of the sequence shown in black and 76 data points from the second half of the sequence shown in blue). (F) Linear correlation between the normalized response to the first echo and the preference index to the second echo of a cascade. Spearman: *r*=−0.3069, ****P<10^−5^, *n*=152 (76 data points from the first half of the sequence shown in black and 76 data points from the second half of the sequence shown in blue).

Because the leading echo of the stimuli covered an echo delay range between 1 and 10 ms (Fig. 1D), neurons tuned to long echo delays (>10 ms) did not respond to the first echo but rather to the second echo of a cascade. For example, by taking a closer look at a multi-unit that predominantly responded to the second echo in the second half of the sequence, it becomes clear that the multi-unit responded poorly to echoes of object B in the single-object sequence (Fig. 5C). Contrastingly, the response to object C was much stronger (red arrow in Fig. 5D). Therefore, it might be not surprising that the response to the second half of the multi-object sequence mostly resembled the response to object C (correlation value=0.72) and only poorly the response to object B (correlation value=0.34; Fig. 5A,C,D). In line with this representative multi-unit, the response strengths to the first echo of an echo cascade positively correlated with the neuronal suppression to the subsequent echo for all multi-units (Spearman: *r*=0.5, *P*<10^−5^, *n*=152 echo–echo pairs; Fig. 5E). Because the neuronal suppression to the subsequent echo increases with the neuronal response to the first echo of a cascade, it is not surprising that the preference of responding to the subsequent echo decreases with an increasing response to the preceding (first) echo (Spearman: *r*=−0.31, *P*<10^−5^, *n*=152 echo–echo pairs; Fig. 5F).

### Neuronal responses to echo cascades at varying echo levels

**Fig. 6.**
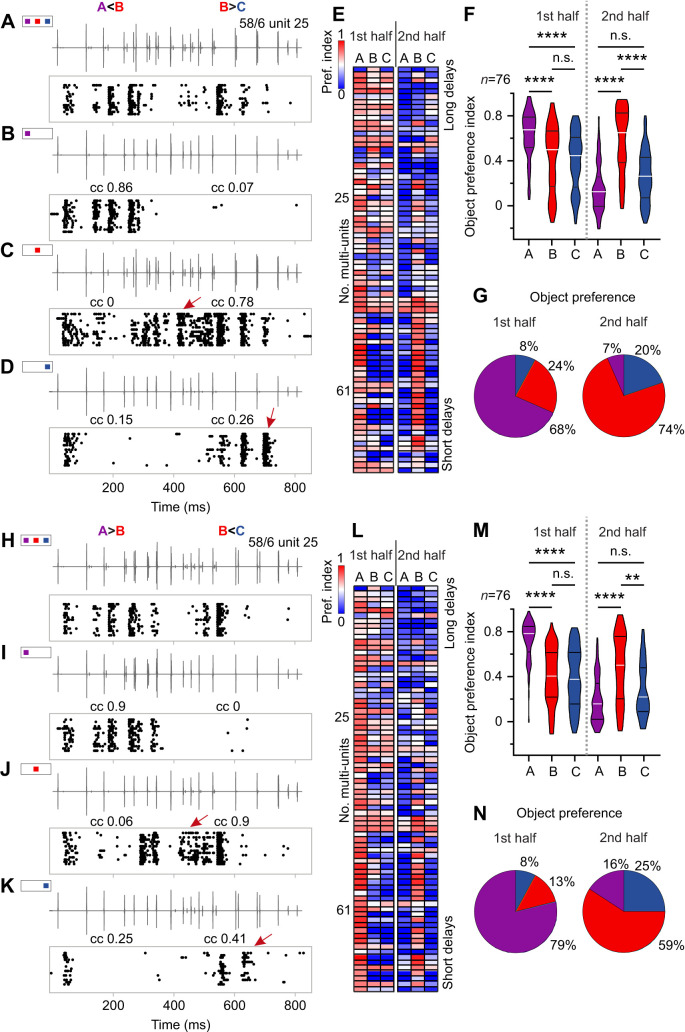
**Cortical neurons predominantly respond to the first echo of a cascade independent of the echo level.** (A–D) Top: oscillograms of the acoustic stimuli. Schemes in the upper left corner indicate the objects whose echoes were present in the corresponding echolocation sequence. Notably, echoes from object B were most intense. Bottom: raster plots visualizing the neural response of one multi-unit. Correlation values (cc) were computed by comparing the neural response to one of the single-object sequences with the response to the multi-object sequence. Correlation values were separately computed for the responses to the first and second half of the sequence. Red arrows indicate the neural responses that were suppressed in the multi-object sequence. (E) Correlation values (preference indices) from the neural response to each single-object sequence (objects A, B, C) and the multi-object sequence for the first and second half of the sequences plotted as colormap. Multi-units are ordered according to their best delay. (F) Violin plots summarizing the object preference indices from 76 multi-units. Friedman test: *P*<10^−5^, Friedman statistic=132.5, *n*=76; Dunn's multiple comparisons test: object A versus B first half: *P*<10^−5^, *Z*=4.77; A versus C first half: *P*<10^−5^, *Z*=4.86; B versus C first half: *P*>0.9999, *Z*=0.087; A versus B second half: *P*<10^−5^, *Z*=7.89; A versus C second half: *P*=0.2565, *Z*=2.39; B versus C second half: *P*<10^−5^, *Z*=5.51. (G) Pie charts visualizing the percentage of object preferences for the first and second half of the sequence (*n*=76 multi-units). (H–N) The same plots as for A–G but when the echo level of object B was lowest within each echo cascade. Statistics for M: Friedman test: *P*<10^−5^, Friedman statistic=138.9, *n*=76; Dunn's multiple comparisons test: object A versus B first half: *P*<10^−5^, *Z*=5.85; A versus C first half: *P*<10^−5^, *Z*=6.16; B versus C first half: *P*>0.9999, *Z*=0.304; A versus B second half: *P*<10^−5^, *Z*=5.16; A versus C second half: *P*>0.9999, *Z*=1.52; B versus C second half: *P*=0.0041, *Z*=3.64.

The results from the multi-object sequences in which echoes were either equally intense (Fig. 3) or the first echo was most intense within a cascade (Fig. S5) (Beetz et al., 2016a) suggest that the leading echo is always predominantly processed in the cortex, as long as the echo delays represented by the leading echo fall in the sensitivity range of the multi-unit. However, what happens if the leading echo of a cascade is fainter than lagging echoes? In nature, this stimulus scenario may indeed occur; for example, when the closest object is smaller than objects in the background. To test the influence of varying echo levels within a cascade more explicitly, we manipulated the echo levels and constructed two multi-object sequences. In one sequence, echoes from object B were most intense (Fig. 1F,G) and for the second sequence, echoes from object B were least intense (Fig. 1H,I) within a cascade. If echo level differences within a cascade affect the neural response, we predicted that modifying echo levels of object B from most to least intense within a cascade would affect the object preference. For example, it is plausible that echoes from object B are intense enough to overcome neural suppression induced by the preceding echo. Against this assumption, even if the second echo of a cascade was more intense than the first echo, most multi-units still responded predominantly to the first echo (Fig. 6). This indicates that varying the echo levels within an echo cascade does not substantially affect the neural response. Instead, the first echo of a cascade is predominantly processed irrespective of its intensity (Fig. 6F,M).

**Fig. 7.**
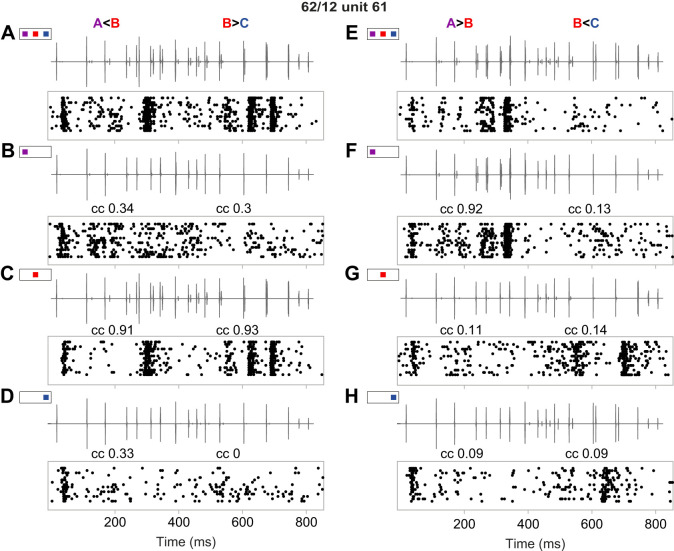
**Example multi-unit where the object preference was affected by the echo intensity.** (A–D) Top: oscillograms of the acoustic stimuli. Schemes in the upper left corner indicate the objects whose echoes were present in the echolocation sequence. Bottom: raster plots visualizing the neural response. Correlation values (cc) indicate the similarity of the neural response to one of the single-object sequences and the response to the multi-object sequences. Correlation values were separately computed for the responses to the first and second half of the sequence. (E–H) The same plots as for A–D but echoes from object B were least intense within an echo cascade. Notably, for the first half of the sequence, the object preference changed from object B (A–D) to object A (E–H) when echoes from object A were more intense than echoes from object B.

Only if the echo was too faint to evoke a clear neural response did the multi-units predominantly process subsequent echoes from a cascade (Fig. 7A–D; Fig. S6A–D). Increasing the level of the first echo, so that it surpassed the multi-unit's response threshold, affected the object preference (Fig. 7E–H; Fig. S6E–H). This was observed in 34% of the multi-units (26 out of 76 multi-units) and may explain the slight increase in the object A preference from 68% to 79% (purple sectors for first half in Fig. 6G,N) when the echo intensity of object A was increased.

In summary, when the first echo of a cascade evokes a neural response, it is followed by a suppression of subsequent echoes. Cortical neurons hence predominantly respond to the first echo that follows a pulse, i.e. echoes from object A in the first half and echoes from object B in the second half of multi-object sequences.

## DISCUSSION

Echolocating bats often encounter echo cascades representing spatial information from multiple objects. Previous studies that focused on echo cascade processing reported that cortical neurons predominantly responded to the first echo following the pulse (Beetz et al., 2016a; Greiter and Firzlaff, 2017). Neural responses to lagging echoes were suppressed in the response to the multi-object sequence. Here, we show that the dominance of processing the leading echo of a cascade is mostly independent of the echo levels. Even when lagging echoes were more intense than the leading one, they could not overcome forward suppression induced by the leading echo (Fig. 5). Only when the intensity of the leading echo dropped below the spike threshold did the neurons respond to the second echo of a cascade (Figs 4 and 6).

The stimulus scenario that we have used here may be biologically plausible considering that the echo level depends not only on the object's distance but also on its reflective surface and size. For example, large or smooth objects evoke more intense echoes than small or rough ones (Firzlaff et al., 2007).

The mild effects of varying echo levels on processing echo cascades are mostly consistent with findings in *Phyllostomus discolor* (Greiter and Firzlaff, 2017), a species that was studied using echo cascades from spatially non-aligned objects (Greiter and Firzlaff, 2017). In the *P. discolor* study, the echolocation sequence simulated a scenario in which the bat laterally passed target 1 before approaching target 2. This means that at a certain lateral distance to target 1, echo levels of target 1 rapidly dropped and became fainter than echoes from target 2. Despite these abrupt changes in the echo level, cortical neurons predominantly responded to the leading echo. Only immediately before passing target 1 did many neurons tend to shift their preference to process target 2. One could argue that echo levels of target 1 were – at that part of the sequence – too faint to evoke a neural response; however, this was excluded when comparing the neural response to the single-object sequence containing echoes from target 1 (Greiter and Firzlaff, 2017). The authors instead explained the shift in target preference as a complex interaction of multiple parameters and not purely based on the echo order or echo levels (Greiter and Firzlaff, 2017). This is contrasted by our present findings in *C. perspicillata*, although we did not systematically test the sensitivity of the multi-units at different echo levels. Our finding that the neural response to the leading echo correlated with a lower response to the lagging echo (when compared with responses obtained in single object sequence, Fig. 4) suggests a strong influence of forward suppression on echo cascade processing. In addition, reducing the level of the leading echo below the multi-unit's response threshold induced a shift in processing echoes from more distant objects (Fig. 6). Altogether, our data suggest that the echo order mostly determines which echo of a cascade is processed, i.e. on a first-come-first-served basis, and that echo levels only affect echo cascade processing, when the leading echo is too faint to evoke a neural response followed by a forward suppression. Preferentially encoding the first echo of a cascade is behaviourally relevant in the context of obstacle avoidance where the most immediate object is the object that a flying bat must negotiate first. Thus, a dominance of keeping track of the position of the most immediate object allows the bat to rapidly negotiate obstacles. Because the bats were anaesthetized in our recordings, the collected data were independent of attentional processes. The dominance of processing the first echo may be smaller when the bat is actively echolocating and focusing on distant targets. However, neural recordings from freely flying bats (*Eptesicus fuscus*) demonstrated that the neurons predominantly processed the first echo following a pulse emission (Kothari et al., 2018), which is comparable to our results from anaesthetized bats.

The present study is based on neural data collected in response to non-overlapping echoes, i.e. every echo was a discrete acoustic event. However, the acoustic environment becomes more complex when echoes are temporally overlapping; for example, when a target is just a few millimetres away from background clutter (Allen et al., 2021). In this case, multiple echoes temporally overlap, thus creating a single echo with a spectral interference pattern composed of notches and peaks (Nordmark, 1960; Pye, 1960, 1961). Depending on the distance between the objects, spectral notches and peaks appear at different frequencies. This means that distance discrimination at the millimetre range is probably based on information from the spectral rather than the temporal domain, such as the echo delay. Importantly, neurons tuned to spectral interference patterns have been described in different bat species (*E. fuscus*: Sanderson and Simmons, 2000, 2002; *P. discolor*: Firzlaff et al., 2006; and *Tadarida brasiliensies*: Macias et al., 2020).

### Auditory stream segregation in the auditory cortex

Based on single-unit recordings in *E fuscus*, Dear et al. (1993) proposed a concurrent representation of multiple objects in the auditory cortex. This hypothesis was inspired by the observation that delay-tuned neurons of *E. fuscus* typically respond with one action potential at a fixed latency to a pulse–echo pair. This precise neural response may allow a concurrent representation of distances to multiple objects within the same neuron. For example, a neuron whose delay-tuning curve covers echo delays from two echoes in a cascade could, because of its phasic response, encode both echoes. In contrast to findings in *E. fuscus*, cortical delay-tuned neurons in *C. perspicillata* show a more tonic and less precise response to pulse–echo pairs, e.g. Fig. 3. Therefore, a neuron that could potentially respond to two echoes of a cascade responds primarily to the leading echo, while the response to the lagging echo is suppressed (Beetz et al., 2016a; Greiter and Firzlaff, 2017).

**Fig. 8.**
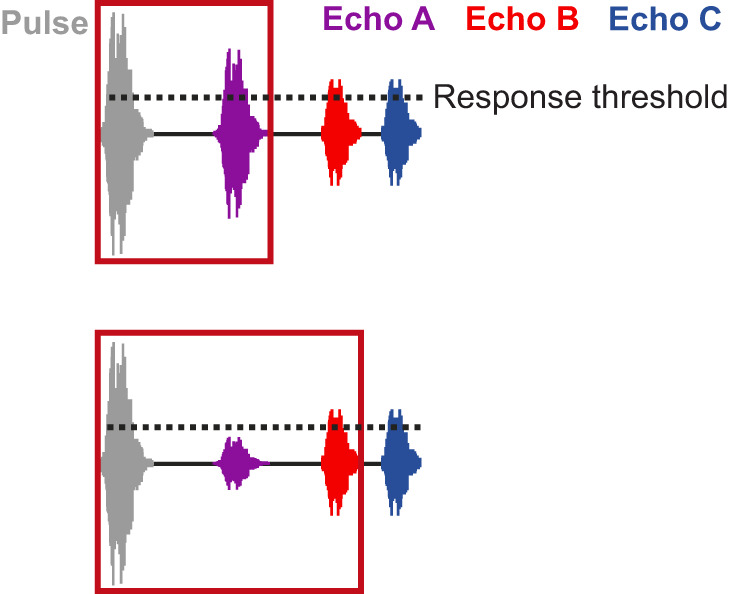
**The ‘time window of sensation’ explains neural responses to echo cascades.** A pulse, even if it is not broadcasted by the bat, opens a ‘time window of sensation’ (indicated in red). The first echo surpassing the neuron's response threshold closes the time window (upper row), rendering the neuron insensitive to lagging echoes. If the leading echo is too faint (lower row), the second echo of a cascade could be processed as long as it is intense enough to surpass the neuron's response threshold. Through neuron-specific spike thresholds, it is possible that multiple objects are processed in parallel in the auditory cortex.

Notably, our results are consistent with the neural time window of sensation (Fig. 7) that was proposed by Roverud and Grinnell (1985) after they trained *N. albiventris* in a distance discrimination task and presented artificial echoes that potentially interfered with the bat's echolocation system. The bats were unable to process distance information correctly only when artificial echoes were presented immediately after pulse emission and before echo arrival. Roverud and Grinnell (1985) concluded that the pulse emission gates the opening of a ‘time window of sensation’ that stays open for ∼27 ms. In that time window, the bat is sensitive to high-frequency acoustic signals that fall within the spectral range of the bat's echolocation signal, which are automatically assigned as an echo. This mechanism only works for distance discrimination in the temporal domain, i.e. when acoustic signals are temporally non-overlapping, which is the case for the search and early approach phases of a target pursuit of an insectivorous bat. The mechanism prevents lagging echoes from being processed because the first high-frequency signal following the pulse closes the time window of sensation. In a previous study (Beetz et al., 2018), we recreated the acoustic scenario of Roverud and Grinnell's (1985) behavioural experiments. We presented echolocation sequences in the absence and presence of an acoustic interferer and noticed that delay tuning was mostly affected when the interferer occurred at pulse–echo pairs representing long echo delays (Beetz et al., 2018). Consistent with these results, the leading echo of a cascade will automatically close the time window of sensation, as long as the echo is intense enough to surpass the spike threshold (Fig. 8). This neural filter avoids the delay-tuned neurons processing distances between objects, a parameter that can be circumscribed as allocentric distance-coding because the distance between objects does not depend on the bat's relative distance to the objects (egocentric distance). Although an allocentric distance coding is important to form a spatial map, we found no hints that delay-tuned neurons of the auditory cortex of *C. perspicillata* encode distances in an allocentric frame of reference. Instead, they purely encode the delay between a pulse and an echo, which represents egocentric distance coding, i.e. the distance between the bat and an object. Notably, examination of auditory object cells recently described in the hippocampus of *E. fuscus* revealed that some neurons encode in egocentric and others in allocentric coordinates (Krishna et al., 2025). The brain region and the underlying mechanisms of the transformation from egocentric distance coding at the cortex level to allocentric distance coding in the hippocampus await discovery.

### Parallel processing of multiple objects

At first glance, the time window of sensation prevents a concurrent representation of multiple objects at the cortex level. How then do bats process lagging echoes, i.e. more distant objects? Recordings of local field potentials from the auditory midbrain revealed modulations to each echo of a cascade as long as the inter-echo intervals were longer than 1 ms (Warnecke et al., 2018). This goes in line with single-unit recordings from the auditory midbrain of bats that were passively listening to multi-object sequences (Beetz et al., 2017). A representation of each echo of a cascade was also observed in local field potentials recorded from the cochlear nucleus, albeit the response variability was greater for late echoes from a cascade than for the leading echo (Simmons et al., 2024). Altogether, the data suggest that echo streams of multiple objects can be processed in parallel up to the midbrain. The preservation of each echo of a cascade disappears at the level of the auditory cortex (Beetz et al., 2016a, 2017), which was suggested to be based on forward suppression (Beetz et al., 2016b), which is much more prominent at the cortex than in the inferior colliculus (Beetz et al., 2017). Our findings indicating that neuronal responses to lagging echoes of a cascade are mostly degraded when the multi-unit responds strongly to the leading echo are consistent with the idea that forward suppression is one of the underlying mechanisms of echo cascade processing. In line with this interpretation is the fact that multi-units whose best delays (i.e. >10 ms) are not represented by the first echo of the cascades tend to respond to the lagging echo and could therefore encode lagging echoes from more distant objects.

Notably, some delay-tuned neurons of the auditory cortex show a non-monotonic response, i.e. the response strength decreases with increasing echo level (Hechavarría and Kössl, 2014). Non-monotonicity in the cortex could ensure that some cortical neurons predominantly respond to faint subsequent echoes while the first echo is too intense to evoke a neural response and hence a neuronal suppression. Therefore, non-monotonic responses could keep the cortex sensitive to faint lagging echoes and hence may allow a concurrent representation of multiple object distances in the cortex of *C. perspicillata*. However, this hypothesis must be tested in future experiments that explicitly test the relationship between non-monotonicity and processing echo cascades in delay-tuned neurons of the auditory cortex.

## Supplementary Material

10.1242/jexbio.252069_sup1 Supplementary information

## References

[JEB252069C1] Allen, K. M., Salles, A., Park, S., Elhilali, M. and Moss, C. F. (2021). Effect of background clutter on neural discrimination in the bat auditory midbrain. *J. Neurophysiol.* 126, 1772-1782. 10.1152/jn.00109.2021 34669503 PMC8794058

[JEB252069C2] Beetz, M. J. and Hechavarría, J. C. (2022). Neural processing of naturalistic echolocation signals in bats. *Front. Neural Circuits* 16, 899370. 10.3389/fncir.2022.899370 35664459 PMC9157489

[JEB252069C3] Beetz, M. J., Hechavarría, J. C. and Kössl, M. (2016a). Cortical neurons of bats respond best to echoes from nearest targets when listening to natural biosonar multi-echo streams. *Sci. Rep.* 6, 35991. 10.1038/srep35991 27786252 PMC5081524

[JEB252069C4] Beetz, M. J., Hechavarría, J. C. and Kössl, M. (2016b). Temporal tuning in the bat auditory cortex is sharper when studied with natural echolocation sequences. *Sci. Rep.* 6, 29102. 10.1038/srep29102 27357230 PMC4928181

[JEB252069C5] Beetz, M. J., Kordes, S., García-Rosales, F., Kössl, M. and Hechavarría, J. C. (2017). Processing of natural echolocation sequences in the inferior colliculus of Seba's fruit eating bat, *Carollia Perspicillata*. *eNeuro* 4, ENEURO.0314-17.2017. 10.1523/ENEURO.0314-17.2017 PMC5729038 29242823

[JEB252069C6] Beetz, M. J., García-Rosales, F., Kössl, M. and Hechavarría, J. C. (2018). Robustness of cortical and subcortical processing in the presence of natural masking sounds. *Sci. Rep.* 8, 6863. 10.1038/s41598-018-25241-x 29717258 PMC5931562

[JEB252069C7] Dear, S. P., Simmons, J. A. and Fritz, J. (1993). A possible neuronal basis for representation of acoustic scenes in auditory-cortex of the big brown bat. *Nature* 364, 620-623. 10.1038/364620a0 8350920

[JEB252069C8] Esser, K. H. and Eiermann, A. (1999). Tonotopic organization and parcellation of auditory cortex in the FM-bat *Carollia perspicillata*. *Eur. J. Neurosci.* 11, 3669-3682. 10.1046/j.1460-9568.1999.00789.x 10564374

[JEB252069C9] Feng, A. S., Simmons, J. A. and Kick, S. A. (1978). Echo detection and target-ranging neurons in auditory-system of bat *Eptesicus-Fuscus*. *Science* 202, 645-648. 10.1126/science.705350 705350

[JEB252069C10] Firzlaff, U., Schornich, S., Hoffmann, S., Schuller, G. and Wiegrebe, L. (2006). A neural correlate of stochastic echo imaging. *J. Neurosci.* 26, 785-791. 10.1523/JNEUROSCI.3478-05.2006 16421298 PMC6675356

[JEB252069C11] Firzlaff, U., Schuchmann, M., Grunwald, J. E., Schuller, G. and Wiegrebe, L. (2007). Object-oriented echo perception and cortical representation in echolocating bats. *PLoS Biol.* 5, 1174-1183. 10.1371/journal.pbio.0050100 PMC1847841 17425407

[JEB252069C12] Fujioka, E., Aihara, I., Watanabe, S., Sumiya, M., Hiryu, S., Simmons, J. A., Riquimaroux, H. and Watanabe, Y. (2014). Rapid shifts of sonar attention by *Pipistrellus abramus* during natural hunting for multiple prey. *J. Acoust. Soc. Am.* 136, 3389-3400. 10.1121/1.4898428 25480083

[JEB252069C13] Gao, L., Balakrishnan, S., He, W. K., Yan, Z. and Müller, R. (2011). Ear deformations give bats a physical mechanism for fast adaptation of ultrasonic beam patterns. *Phys. Rev. Lett.* 107, 214301. 10.1103/PhysRevLett.107.214301 22181884

[JEB252069C14] Greiter, W. and Firzlaff, U. (2017). Echo-acoustic flow shapes object representation in spatially complex acoustic scenes. *J. Neurophysiol.* 117, 2113-2124. 10.1152/jn.00860.2016 28275060 PMC5454466

[JEB252069C15] Grinnell, A. D. (1963). Neurophysiology of audition in bats - temporal parameters. *J. Physiol. Lond.* 167, 67-96. 10.1113/jphysiol.1963.sp007133 13950554 PMC1359485

[JEB252069C16] Hagemann, C., Esser, K. H. and Kössl, M. (2010). Chronotopically organized target-distance map in the auditory cortex of the short-tailed fruit bat. *J. Neurophysiol.* 103, 322-333. 10.1152/jn.00595.2009 19906883

[JEB252069C17] Hagemann, C., Vater, M. and Kössl, M. (2011). Comparison of properties of cortical echo delay-tuning in the short-tailed fruit bat and the mustached bat. *J Comp. Physiol. A* 197, 605-613. 10.1007/s00359-010-0530-8 20446089

[JEB252069C18] Hechavarría, J. C. and Kössl, M. (2014). Footprints of inhibition in the response of cortical delay-tuned neurons of bats. *J. Neurophysiol.* 111, 1703-1716. 10.1152/jn.00777.2013 24478161

[JEB252069C19] Hechavarría, J. C., Macías, S., Vater, M., Voss, C., Mora, E. C. and Kössl, M. (2013). Blurry topography for precise target-distance computations in the auditory cortex of echolocating bats. *Nat. Commun.* 4, 2587. 10.1038/ncomms3587 24107903

[JEB252069C20] Kothari, N. B., Wohlgemuth, M. J. and Moss, C. F. (2018). Dynamic representation of 3D auditory space in the midbrain of the free-flying echolocating bat. *eLife* 7, e29053. 10.7554/eLife.29053 29633711 PMC5896882

[JEB252069C21] Krishna, A., Yin, X., Yu, C., Skandalis, D. A., Lee, H. and Moss, C. F. (2025). Auditory object representation in the bat hippocampus. *Curr. Biol.* 35, 4311-4320.e4. 10.1016/j.cub.2025.07.044 40812304 PMC12355007

[JEB252069C22] Linnenschmidt, M. and Wiegrebe, L. (2016). Sonar beam dynamics in leaf-nosed bats. *Sci. Rep.* 6, 29222. 10.1038/srep29222 27384865 PMC4935842

[JEB252069C23] Macias, S., Bakshi, K., Garcia-Rosales, F., Hechavarria, J. C. and Smotherman, M. (2020). Temporal coding of echo spectral shape in the bat auditory cortex. *PLoS Biol.* 18, e3000831. 10.1371/journal.pbio.3000831 33170833 PMC7678962

[JEB252069C24] Martin, L. M., García-Rosales, F., Beetz, M. J. and Hechavarría, J. C. (2017). Processing of temporally patterned sounds in the auditory cortex of Seba's short-tailed bat, *Carollia perspicillata*. *Eur. J. Neurosci.* 46, 2365-2379. 10.1111/ejn.13702 28921742

[JEB252069C25] Moss, C. F. and Surlykke, A. (2010). Probing the natural scene by echolocation in bats. *Front. Behav. Neurosci.* 4, 33. 10.3389/fnbeh.2010.00033 20740076 PMC2927269

[JEB252069C26] Neuweiler, G. (1990). Auditory adaptations for prey capture in echolocating bats. *Physiol. Rev.* 70, 615-641. 10.1152/physrev.1990.70.3.615 2194220

[JEB252069C27] Nordmark, J. (1960). Perception of distance in animal echo-location. *Nature* 188, 1009-1010. 10.1038/1881009a0 13729545

[JEB252069C28] Pye, J. D. (1960). A theory of echolocation by bats. *J. Laryngol. Otol.* 74, 718-729. 10.1017/S0022215100057170 13738575

[JEB252069C29] Pye, J. D. (1961). Perception of distance in animal echolocation. *Nature* 190, 362-363. 10.1038/190362a0 13729546

[JEB252069C30] Roverud, R. C. and Grinnell, A. D. (1985). Echolocation sound features processed to provide distance information in the CF/FM Bat, *Noctilio-albiventris* - evidence for a gated time window utilizing both CF and FM components. *J. Comp. Physiol. A* 156, 457-469. 10.1007/BF00613970

[JEB252069C31] Sanderson, M. I. and Simmons, J. A. (2000). Neural responses to overlapping FM sounds in the inferior colliculus of echolocating bats. *J. Neurophysiol.* 83, 1840-1855. 10.1152/jn.2000.83.4.1840 10758096

[JEB252069C32] Sanderson, M. I. and Simmons, J. A. (2002). Selectivity for echo spectral interference and delay in the auditory cortex of the big brown bat *Eptesicus fuscus*. *J. Neurophysiol.* 87, 2823-2834. 10.1152/jn.00628.2001 12037185

[JEB252069C33] Seibert, A. M., Koblitz, J. C., Denzinger, A. and Schnitzler, H. U. (2013). Scanning behavior in echolocating common pipistrelle bats (*Pipistrellus pipistrellus*). *PLoS ONE* 8, e60752. 10.1371/journal.pone.0060752 23580164 PMC3620330

[JEB252069C34] Simmons, A. M., Warnecke, M. and Simmons, J. A. (2024). Microseconds-level coding of echo delay in the auditory brainstem of an FM-echolocating bat. *J. Neurophysiol.* 132, 2012-2022. 10.1152/jn.00305.2024 39570280 PMC11687828

[JEB252069C35] Suga, N. and O'Neill, W. E. (1979). Neural axis representing target range in the auditory-cortex of the mustache bat. *Science* 206, 351-353. 10.1126/science.482944 482944

[JEB252069C36] Sullivan, W. E. (1982). Neural Representation of target distance in auditory-cortex of the echolocating bat *myotis-lucifugus*. *J. Neurophysiol.* 48, 1011-1032. 10.1152/jn.1982.48.4.1011 7143030

[JEB252069C37] Surlykke, A., Ghose, K. and Moss, C. F. (2009a). Acoustic scanning of natural scenes by echolocation in the big brown bat, *Eptesicus fuscus*. *J. Exp. Biol.* 212, 1011-1020. 10.1242/jeb.024620 19282498 PMC2726860

[JEB252069C38] Surlykke, A., Pedersen, S. B. and Jakobsen, L. (2009b). Echolocating bats emit a highly directional sonar sound beam in the field. *Proc. R. Soc. B Biol. Sci.* 276, 853-860. 10.1098/rspb.2008.1505 PMC2664374 19129126

[JEB252069C39] Warnecke, M., Macías, S., Falk, B. and Moss, C. F. (2018). Echo interval and not echo intensity drives bat flight behavior in structured corridors. *J. Exp. Biol.* 221, jeb191155. 10.1242/jeb.191155 30355612

[JEB252069C40] Wohlgemuth, M. J., Kothari, N. B. and Moss, C. F. (2016). Action enhances acoustic cues for 3-D target localization by echolocating bats. *PLoS Biol.* 14, e1002544. 10.1371/journal.pbio.1002544 27608186 PMC5015854

