# Supplementary material for: First come, first served: neuronal processing of multi-echo streams in the auditory cortex of echolocating bats
Source: J Exp Biol. 2026 May 28;229(10):jeb252069. doi: 10.1242/jeb.252069 (PMC13286348; doi:10.1242/jeb.252069)
Supplement: Supplementary information [file jexbio-229-252069-s1.pdf]

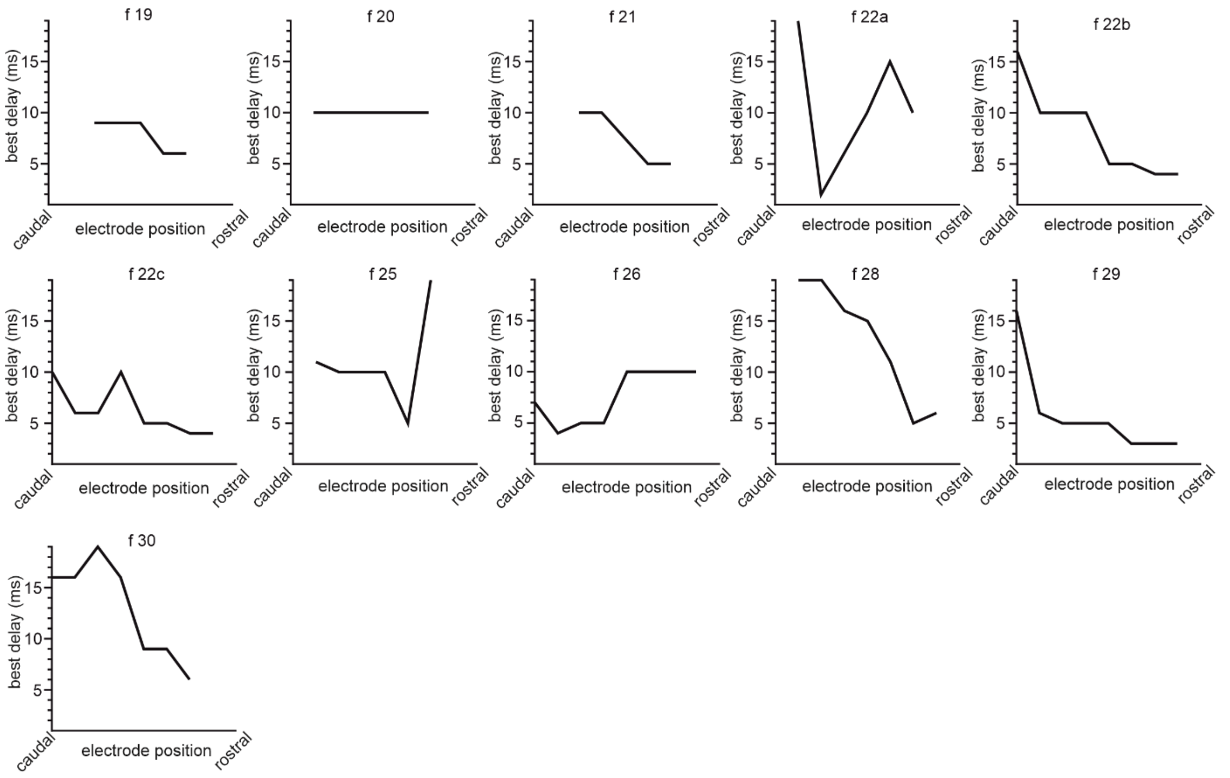

Fig. S1. Chronotopy along the rostral-caudal axis of the cortex. Best delays plotted against the electrode position along the rostral-caudal axis of the cortex for each experiment. ID above each plot indicates the individual bat.

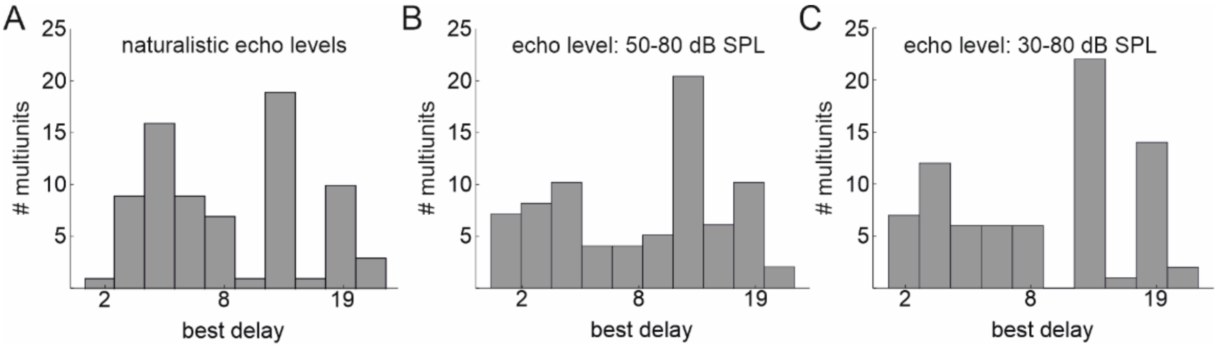

Fig. S2. Best delays measured at different echo levels. A: Distribution of best delays of all 76 multi-units measured in response to a single-object sequence in which echo levels varied naturally. Echo levels are represented as brown dots in figure S5B. **B**: Distribution of best delays of all 76 multi-units measured in response to a single-object sequence in which echo levels varied between 50- and 80-dB SPL. Levels of each echo are represented as brown dots in figure 1G. **C**: Distribution of best delays of all 76 multi-units measured in response to a single-object sequence in which echo levels varied between 30- and 80-dB SPL. Levels of each echo are represented as brown dots in figure 1I.

Journal of Experimental Biology • Supplementary information

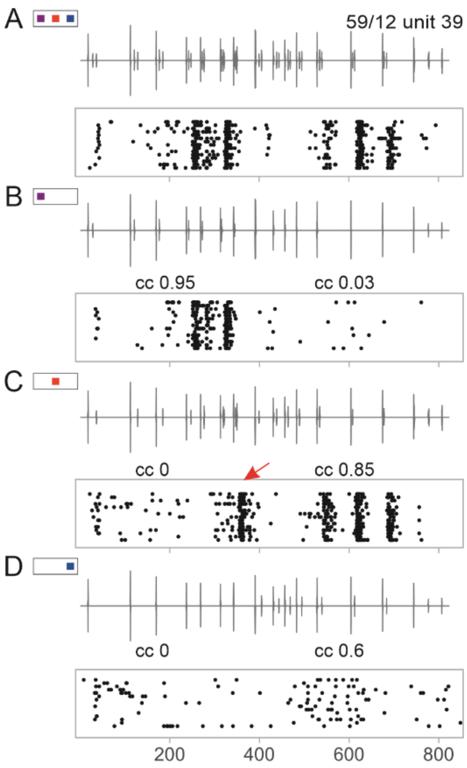

Fig. S3. Response of an exemplary multi-unit to multi- and single-object sequences. A-D: *Top*: Oscillograms of the acoustic stimuli. Schemes in the upper left corner indicate the objects whose echoes were present in the echolocation sequence. *Bottom*: Raster plots visualizing the neural response of the multi-unit. Correlation values (cc) indicate the similarity of the neural response to one of the single-object sequence and the response to the multi-object sequence shown in A. Correlation values were separately computed for the responses to the first and second half of the sequence. Red arrow signals the neural response to object B that was suppressed in the multi-object sequence due to the presence of echoes from object A.

Journal of Experimental Biology • Supplementary information

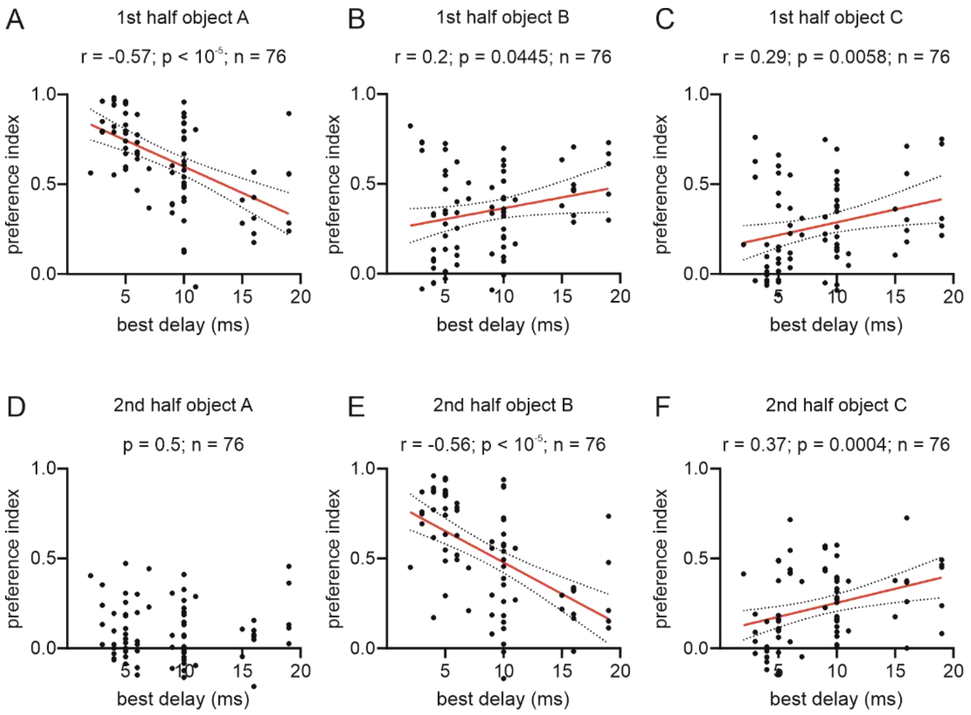

Fig. S4. Correlation between the preference indices and the multi-units' best delays. A-C: Correlations for the first half of the sequence where echoes belonging to object A were the first echoes of a cascade. Each data point represents a multi-unit. **D-F:** Correlations for the second half of the sequence where echoes belonging to object B were the first echoes of a cascade. Spearman statistics: A: r = -0.57; p < 10-5; n = 76 multi-units; B: r = 0.2; p = 0.0445; n = 76 multi-units; C: r = 0.29; p = 0.0058; n = 76 multi-units; D: r = - 0.0008464; p = 0.4971; n = 76 multi-units; E: r = -0.56; p < 10-5; n = 76 multi-units; F: r = 0.37; p = 0.0004; n = 76 multi-units.

Journal of Experimental Biology • Supplementary information

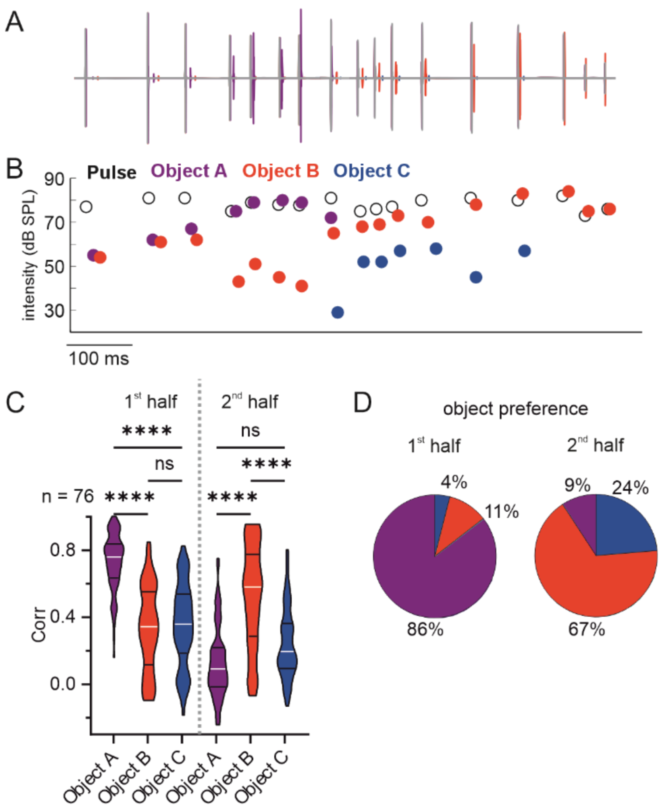

Fig. S5. Results obtained with echolocation sequences representing naturalistic echo levels. A: Oscillogram of a multi-object sequence with naturalistic pulse echo levels. **B**: Pulse and echo levels and their time points are shown. **C**: Violin plots summarizing the object preference indices from 76 multi-units. Friedman test: $p < 10^{-5}$, Friedman statistic = 162.9, n = 76; Dunn's multiple comparisons test: A vs B 1st half: $p < 10^{-5}$, Z = 7.11; A vs C 1st half: $p < 10^{-5}$, Z =7.11; B vs C 1st half: $p > 0.9999$, Z = 0.000; A vs B 2nd half: $p < 10^{-5}$, Z = 6.417; A vs C 2nd half: $p > 0.9999$, Z = 1.691; B vs C 2nd half: $p < 10^{-5}$, Z = 4.726. **D**: Pie charts visualizing the percentage of object preferences for the first half of the sequence (*left*) and second half of the sequence (*right*) for 76 multi-units.

Journal of Experimental Biology • Supplementary information
